# Draft Genome Sequence of the Bacteriocin-Producing Strain *Enterococcus faecium* M3K31, Isolated from Griffon Vultures (*Gyps fulvus* subsp. *fulvus*)

**DOI:** 10.1128/genomeA.00055-16

**Published:** 2016-03-24

**Authors:** Sara Arbulu, Cyril Frantzen, Christopher T. Lohans, Luis M. Cintas, Carmen Herranz, Helge Holo, Dzung B. Diep, John C. Vederas, Pablo E. Hernández

**Affiliations:** a Departmento de Nutrición, Bromatología y Tecnología de los Alimentos, Facultad de Veterinaria, Universidad Complutense de Madrid, Madrid, Spain; b Department of Chemistry, Biotechnology and Food Science, Norwegian University of Life Sciences, Ås, Norway; c Department of Chemistry, University of Alberta, Edmonton, Alberta, Canada

## Abstract

*Enterococcus faecium* M3K31 is a bacteriocinogenic lactic acid bacterium (LAB) isolated from griffon vulture (*Gyps fulvus* subsp. *fulvus*) feces. The draft genome sequence of this strain provides genetic data that support its biotechnological potential.

## GENOME ANNOUNCEMENT

The enterococci are a diverse group of Gram-positive gastrointestinal (GI) tract colonizers recognized as microorganisms of applied, regulatory, and biotechnological interest (1–3). *Enterococcus faecium* M3K31 is a bacteriocinogenic lactic acid bacterium (LAB) isolated from the feces of griffon vultures (*Gyps fulvus* subsp. *fulvus*), with high antimicrobial activity against *Listeria* spp., and it is the producer of the bacteriocin enterocin HF (EntHF) (4). Genomic DNA from *E. faecium* M3K31 was purified using the DNeasy blood and tissue kit (Qiagen, Valencia, CA, USA) and sequenced by using a MiSeq platform (Illumina, Inc., San Diego, CA, USA) at the DNA High-Throughput Sequencing and Genotypic Unit of the University of Illinois at Urbana-Champaign (IL). The shotgun DNAseq library was prepared with the Kapa library preparation kit (Kapa Biosystems, Inc., Wilmington, MA). The library was quantitated by quantitative PCR (qPCR) and sequenced for 251 cycles using a MiSeq sequencing kit version 2. Reads were quality filtered using Nesoni (version 0.130; P. Harrison, 2015) and *de novo* assembled using SPAdes (version 3.5) (5). Contigs <1,000 bp and with coverage <5-fold were removed. Coding DNA sequences (CDSs) were predicted and annotated using the RAST (http://rast.nmpdr.org/) server (6).

The draft genome of *E. faecium* M3K31 consists of 70 contigs, for a total of 2,722,557 bp, with a G+C content of 38.1%. The largest contig was 245,388 bp, and the smallest contig was 1,062 bp. The total number of CDSs was 2,687, and the number of RNAs was 79. *In silico* analysis of the draft genome sequence with the BAGEL3 software (http://bagel2.molgenrug.nl/) (7) confirmed the presence of the EntHF biosynthetic cluster (GenBank accession numbers P86183 and KJ442693), the enterocin P structural and immunity genes (8), and the gene encoding a bacteriocin (GenBank accession no. ELB21426), which resembles (85% identity) a putative peptide named SRCAM 602 (GenBank accession no. P86393) proposed to be produced (9) but not encoded by *Paenibacillus polymyxa* NRRL-30509 (10). The structural genes related to each bacteriocin were found in three different contigs. The rifampin resistance (*rpoB*) gene was detected, but no other relevant antibiotic resistance genes or genes associated with virulence factors related to invasiveness and disease severity were identified (11). Screening of the whole genome of *E. faecium* M3K31 confirmed the absence of the following virulence or virulence-associated factors: insertion sequence (IS*16*), the enterococcal surface protein (*esp*), and the putative glycosyl hydrolase (*hyl*) virulence genes, hence meeting the European Food Safety Authority (EFSA) requirements for the potential safe use of this enterococcal strain as a feed additive (12). One clustered regularly interspaced short palindromic repeat (CRISPR) array, considered a barrier to foreign DNA uptake, was identified using CRISPRfinder (13). The availability of this draft genome may strengthen the value of the bacteriocin-producing *E. faecium* M3K31 as a potentially useful natural food preservative and therapeutic for human and veterinary applications, and as a potential probiotic for use in animal nutrition.

### Nucleotide sequence accession numbers.

This whole-genome shotgun project has been deposited at DDBJ/EMBL/GenBank under the accession no. LAXK00000000. The version described in this paper is the first version, LAXK01000000.
